# Impact of extended sinus surgery on allograft infection, allograft function and overall survival in cystic fibrosis lung transplant recipients

**DOI:** 10.1007/s00405-023-08028-3

**Published:** 2023-05-30

**Authors:** Manuel Meier, Macé M. Schuurmans, Domenic Vital, Ilhan Inci, David Holzman, Michael B. Soyka

**Affiliations:** 1 Department of Otorhinolaryngology, University Hospital Zurich USZ, Zurich, Switzerland; 2 Department of Pulmonology, University Hospital Zurich USZ, Zurich, Switzerland; 3 Center for Head and Neck Surgery AG, Hirslanden Clinic Cham, Cham, Switzerland; 4 FEBTS, FCCP, University of Zurich, Zurich, Switzerland; 5 Thoracic Surgery, Hirslanden Clinic Zurich, Zurich, Switzerland; 6 Faculty of Medicine, University of Zurich, Zurich, Switzerland

**Keywords:** Cystic fibrosis, Lung transplantation, Extended sinus surgery, Infectious events, Chronic allograft dysfunction

## Abstract

**Background:**

Studies investigating the impact of sinus surgery for cystic fibrosis (CF) patients performed early after lung transplantation (Ltx) are scarce. Recent studies evaluating frequency of respiratory infections and graft outcomes are not available.

**Objectives/hypothesis:**

To determine whether there is a difference in allograft infection, allograft function and overall survival among CF lung transplant recipients with and without concomitant sinus surgery.

**Study design:**

Retrospective single-center study.

**Methods:**

We examined 71 CF patients who underwent Ltx between 2009 and 2019 at our center. Fifty-nine patients had sinus surgery before or/and after transplantation and twelve did not undergo sinus surgery. We assessed the survival, the diagnosis of chronic allograft dysfunction (CLAD) and all elevated (> 5 mg/l) c-reactive protein episodes during the observed period. The infectious events of the upper and lower airways were categorized in mild infections (5–15 mg/l CRP) and severe infections (> 15 mg/l CRP).

**Results:**

There was no difference in the long-time overall survival (*p* = 0.87) and no benefit in the short-term survival at 4 year post-transplant (*p* = 0.29) in both groups. There was no difference in both groups concerning CLAD diagnosis (*p* = 0.92). The incidence of severe upper and lower airway infections (CRP > 15 mg/l) was significantly decreased in the sinus surgery group (*p* = 0.015), whereas in mild infections there was a trend to decreased infections in the sinus surgery group (*p* = 0.056).

**Conclusions:**

CF patients undergoing Ltx benefit from extended endoscopic sinus surgery (eESS) in terms of frequency of severe infectious events of the upper and lower airways. There was no difference in overall survival and frequency of CLAD in the two groups.

## Introduction

CF is the most common autosomal recessive disorder with a frequency of 1–2500 in Caucasians. More than 2000 mutations in the responsible cystic fibrosis transmembrane regulator (CFTR) gene have been detected. Dysfunction of that gene causes pathological changes in secretory cells of different organs, such as lungs, paranasal sinuses, pancreas, liver and reproduction organs. Chronic infections of the lung with certain bacteria (such as *P. aeruginosa* (PA), *S. aureus* (SA), *methicillin-resistant SA* (MRSA), *S. maltophilia*, *A. xylosoxidans*) are one of the main concerns in treating CF patients and contribute to chronic decline in lung function [1, 2]. Ltx is the ultimate only life-prolonging therapy for CF patients with end stage CF-related lung disease. The lung transplant program at the Zurich University Hospital was established in 1992. Since then, more than 550 children and adults with end stage CF disease have received a Ltx at our center. Since the beginning of the program there have been various improvements in short- and long-term post-transplant treatment. The better surgical techniques, organ preservation, intensive-care management and the rigorous post-transplant treatment of airway infections, sinus surgery, routine nasal care, long-term macrolide antibiotics and extracorporeal photopheresis in selected patients seem to have a positive impact on allograft function and patient survival [3]. Nevertheless, late allograft dysfunction continues to be a major issue in the follow-up of these patients. The burden of care for the CF lung transplant recipients is high and includes regular local care of sinuses, routine clinical and diagnostic check-ups. Neglecting the meticulous daily nasal care program frequently results in local and systemic complications sometimes associated with hospitalization for prolonged intravenous antibiotic treatment of upper and lower airways (UAW/LAW) infections [4]. To minimize the risk of chronic infections and colonization compromising post-transplant allograft function, all patients at our institution are treated with empirical and targeted antibiotics for at least 4 weeks after Ltx targeting the previously detected bacteria. As soon as the patients have recovered sufficiently from transplantation (beginning 3–4 week post-transplant), we perform eESS consisting of endoscopic fronto-spheno-ethmoidectomy and strict post-surgery treatment consisting of nasal douching with isotonic saline solution to prevent re-colonization of the sinuses as described earlier [4]. This concept was recently supported by a study of Johnson et al. who showed that early endoscopic sinus surgery interventions improve pulmonary function and attenuated pulmonary exacerbations [5]. Up to 100% of CF patients show radiological or clinical signs of chronic rhinosinusitis (CRS) with a majority of patients having an aplasia or hypoplasia of the frontal and sphenoid sinuses. In general CF patients show a high amount of anatomical sinonasal variations [6]. Based on these findings, we have, therefore, suggested that eESS is indicated in almost all lung transplant recipients with the underlying diagnosis of CF. Patients with significant re-colonization of the nasal and sinus cavities with clinical evidence suggesting LAW colonization are considered for revision surgery as well. Although previous evaluations of our practice have shown encouraging results, we were interested to see if despite various advances in the anti-infective and local management of our patients, eESS is still beneficial for our patients in recent years. In contrast to our previous studies, where we focused on the microbial colonization, we now aimed to analyze if there is a difference in infectious events, chronic allograft dysfunction and overall survival in Ltx patients undergoing eESS at the time of transplantation as compared to CF patients not undergoing eESS post-transplant.

## Methods

We analyzed all CF patients undergoing Ltx at our institution between 01.01.2009 and 01.01.2019. We screened 77 patients of which 71 were eligible for the study. Exclusion criteria were recipients < 18 years of age (2 patients) and death within 4 months after transplantation (4 patients). The mean follow-up of the whole cohort was 68 ± 33 months. Two patients were lost to follow-up concerning infectious events but not regarding to overall survival (change of primary hospital for follow-up visits). One patient was re-transplanted after 4 years and 2 months. All patients were assessed prior to Ltx with a recent CT scan of the paranasal sinuses. If the patients had not had eESS yet or showed any residual cells after surgery, which may compromise the reliable drainage by rinsing of the nose and sinuses and thus allow a potential reservoir of mucus, revision surgery was indicated. All patients underwent ear–nose–throat (ENT) exams prior to Ltx, where we assessed the necessity for eESS after Ltx. Extended sinus surgery was recommended in all but one patient (absence of sinonasal disease in CT scan and no CRS symptoms). Surgery in these patients includes radical fronto-spheno-ethmoidectomy, with at least a Draf 2a procedure on both sides, if pneumatization of the respective sinus was present. Remaining septae were drilled with a high speed diamond drill. No median drainage procedure was necessary. After transplantation, the treatment plan was changed for some of these lung transplant recipients for several reasons and we referred to these patients as the non-eESS group. The reasons were: in three patients a colonization with BCC (Burkholderia cepacia) of the lung or/and the sinuses led to the decision of watchful waiting. All other patients included in the non-eESS group were oligosymptomatic and the treatment team decided not to perform eESS despite the initial indication or the patients were not willing to undergo another surgery shortly after Ltx in agreement with the treatment team. Post-transplant allograft function was regularly assessed and defined at our transplantation center and classified in accordance with the international criteria for CLAD diagnosis and grading. Infectious events such as UAW and LAW infections were screened using high-sensitivity c-reactive protein (CRP) measurements with a cutoff of > 5 mg/l. If we detected such an episode of an elevated CRP we correlated the blood test with the patients symptoms, clinical assessment and diagnostic work-up (blood cultures, imaging studies) and included all episodes in our analysis if the diagnosis of an UAW or LAW infection was made. Due to immunosuppression, the CRP-value in local or systemic infections is much lower in solid organ recipients than in the normal population and is a valid marker in diagnosing infections in that population and an established algorithm at our center [7]. We classified all episodes in mild (CRP > 5–14.9 mg/l) and severe (> 15 mg/l) infections. This has clinical relevance, because in mild infections, oral antibiotic treatment is mostly on an outpatient basis, whereas severe infections are treated consistently on an inpatient basis with intravenous antibiotics. All patient information is documented in our clinical information system. The ethical committee of Zurich (Kantonale Ethikkommission Zurich) approved this retrospective study (BASEC-Nr. 2020-02032).

Descriptive statistics were performed with the Fisher-exact-test for categorical data and the Mann–Whitney *U* test for continuous variables, utilizing SPSS (IBM^®^) Version 26. The Kaplan–Meier method was used to estimate overall survival and CLAD-free-survival and significance testing was performed with log rank test. For the survival analysis, we used the Fisher-exact-test and Chi^2^-test. All tests of significance for the infectious events were calculated with an unpaired *t* test with a *p* value of < 0.05 considered significant. Two-sided tests were performed.

**Fig. 1:**
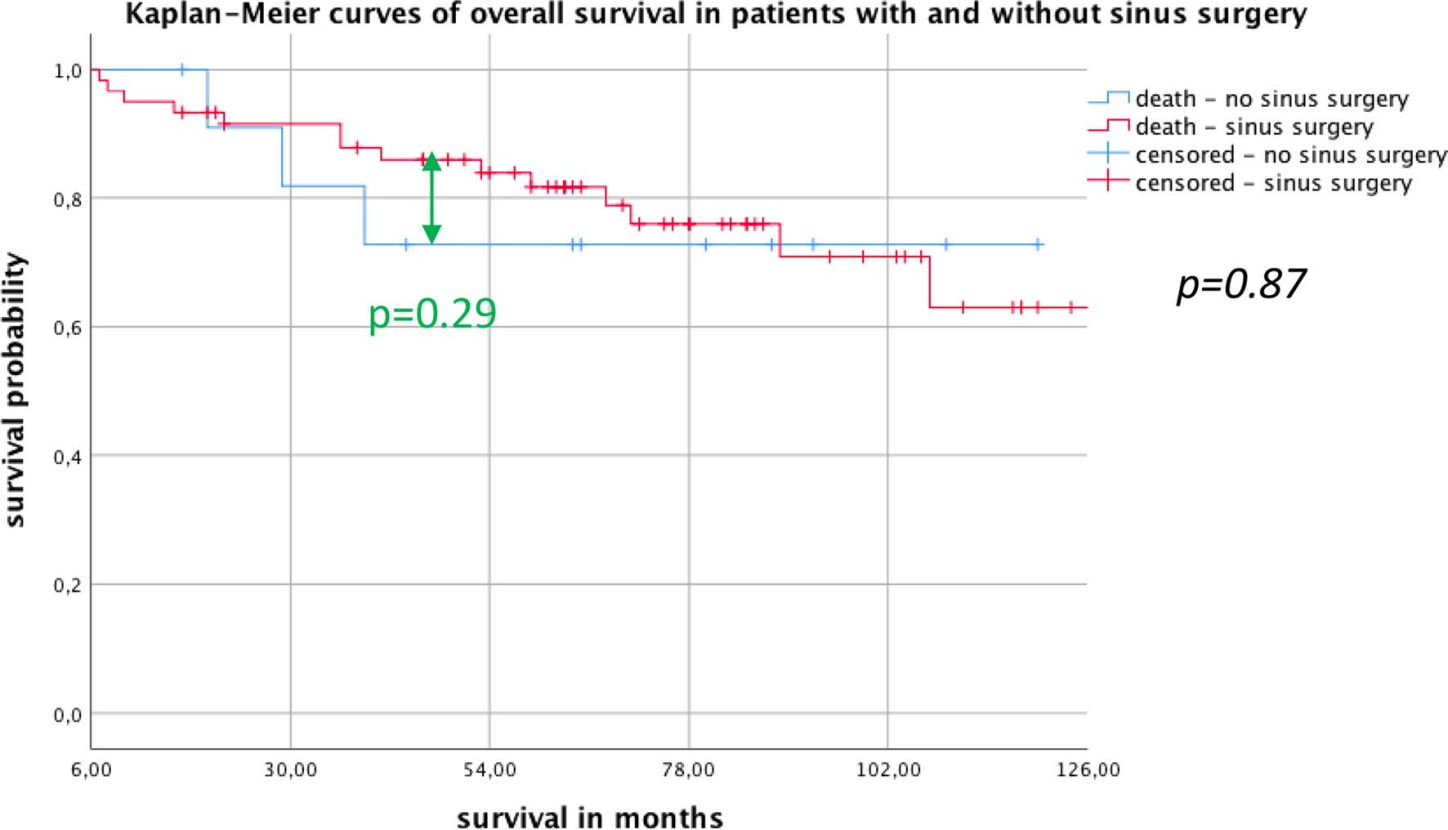
Overall survival

## Results

**Fig. 2:**
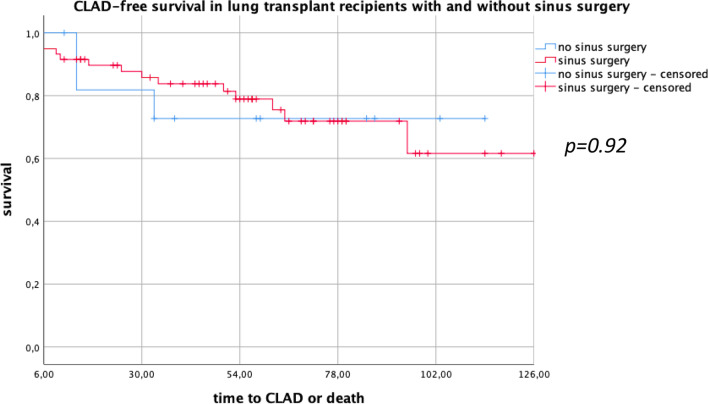
CLAD-free survival

**Fig. 3:**
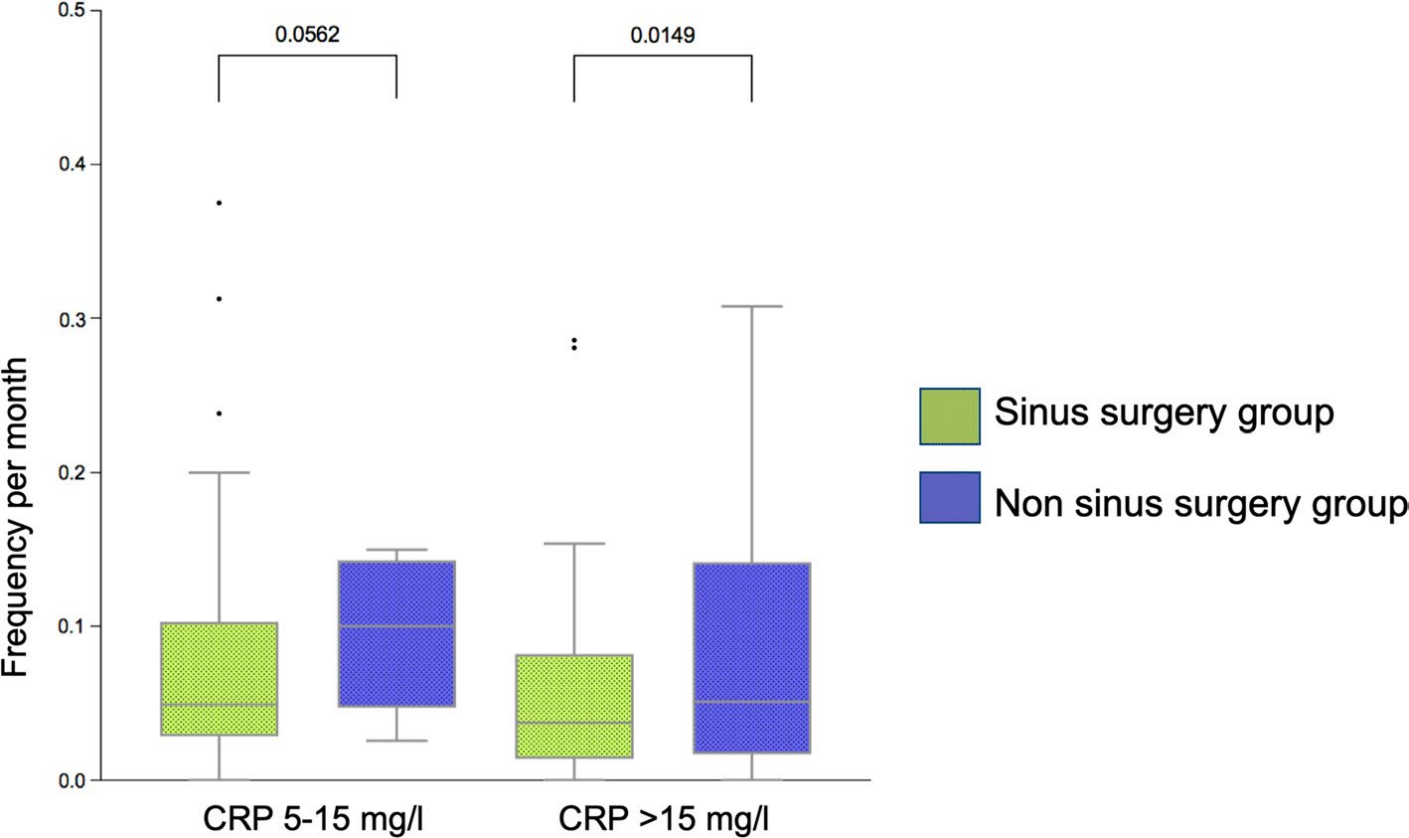
Mild and severe infection episodes

Of all patients (*N* = 71), 59 (83%) underwent sinus surgery before (*N* = 9, 15%) or after Ltx (*N* = 42,71%). Eight patients (14%) had sinus surgery before and after Ltx. Twelve (17%) patients were included in the non-sinus surgery group. The study population consisted of 32 (45%) males and 39 (55%) females. The mean age was 29.4 ± 9.4 years in the eESS group and 25.9 ± 8.8 years in the non-sinus surgery group (*p* = 0.36). The mean time between Ltx and eESS in the above-mentioned 59 patients was 72.9 ± 93.3 days. F508 homozygotes genotypes were dominant in both groups with 36 patients (50%) in the eESS group and 9 patients (13%) in the non-sinus surgery group. Demographic details are shown in Table 1. In the overall survival analysis, there was no significant difference between both groups (*p* = 0.8). The short-term survival analysis at 4 years showed no significance between groups (*p* = 0.29) (Fig. 1). In the CLAD-free survival we observed no significant difference between both groups (*p* = 0.92) (Fig. 2). Evaluating mild infectious events, we found no significant difference in frequency of infections of the LAW/UAW but a trend to fewer infections in the eESS group with an average of 0.076 ± 0.073 events per month compared to 0.13 ± 0.16 events per month in the non-sinus surgery group (*p* = 0.056). Considering severe infections with CRP > 15 mg/l and necessity for inpatient treatment for intravenous antibiotics we see a significant difference of 0.057 ± 0.063 events per month in the eESS group compared to 0.189 ± 0.395 events per month in the non-sinus surgery group (*p* = 0.015) (Fig. 3).

**Table 1:** Patient characteristics

	Extensive sinus surgery group	Non-sinus surgery group	*p* value
Patients (% of total)	59 (83%)	12 (17%)	
Age ± SD (years)	29.4 ± 9.4	25.9 ± 8.8	0.36
BCC colonization	3	2	
Gender			
Male (%)	29 (41%)	3 (4%)	0.20
Female (%)	30 (42%)	9 (13%)	0.20
Genotypes			
dF508 homozygotes (%)	36 (50%)	9 (13%)	0.52
Heterozygot/unknown (%)	23 (32%)	3 (4%)	0.52
Sinus surgery			
Before (%)	9 (15%)	–	
After (%)	42 (71%)	–	
Before and after (%)	8 (14%)	–	
Mean time lung transplantation to sinus surgery (d)	72.9 ± 93.3		
Mean survival ± SD (m)	68.4 ± 33.3	64.0 ± 34.7	0.85

## Discussion

In this retrospective study, a statistically significant and clinically relevant benefit in infectious events was documented for extensive sinus surgery in CF recipients who underwent Ltx. No benefit could be shown for short- and long-term survival and no benefit was shown for the frequency of chronic allograft dysfunction (CLAD-free survival).

The literature on the potential benefit of ESS for long-term survival after lung transplantation is scarce. Leung et al. assessed a heterogeneous cohort of 87 patients undergoing ESS prior to lung transplantation and could not report a difference in recolonization of the allograft nor a benefit in survival compared to other transplant centers not performing ESS pre-transplant [8]. In contrast, our study group showed in different studies a potential benefit of eESS on the survival after Ltx which included a meticulous postoperative nasal care regimen to maintain clean paranasal sinuses. We could show a benefit in overall survival, if a colonialization of the graft and the sinuses with PA could be avoided, which was achieved by eESS and the above-mentioned nasal care [4, 9, 10]. However, no direct comparison of eESS operated and non-operated patients was made in our previous studies. In our current retrospective analysis, there was no difference in overall survival and no benefit in short-term survival at 4 years. There seems to be a difference in the short-term survival in the groups 4 years after Ltx, which could be of high relevance for the single patient because of the expected survival time after Ltx [3]. Due to the small numbers in the groups we were not able to show any significance in the short-term survival. Therefore, we are not able to confirm a large benefit of eESS for short- and long-term overall survival with the present data. In contrast to our previous results, we now did not assess the difference in overall survival in perspective of PA colonization but focused on the difference between surgical and conservative approach. Only focusing on the missing survival benefit our data implies a new, revised approach to the present standard of care and to possibly chose eESS more reluctantly in this specific group of patients.

Reduction of CLAD is of great interest. PA plays a major role in the early development of bronchiolitis obliterans syndrome (BOS), the most frequent form of CLAD [11]. Therefore, the necessity to prevent recolonization of the allograft is crucial. As shown before, the early eradication of reservoirs for PA with eESS and daily nasal rinsing reduces allograft infections in Ltx recipients with CF and has a positive impact on post-transplant survival and the development of chronic allograft dysfunction [10]. Our results did not show a difference in CLAD. Considering that there are different studies supporting the concept of ESS to eradicate PA, preventing recolonization of the allograft and resulting in better lung function outcomes, we must reflect on possible bias and confounders in our data, further discussed in limitations of our study [9, 11, 12].

One corner stone to prevent CLAD, as mentioned before, is to prevent allograft infections. The regular follow-up at the lung transplantation center aims to prevent and, if necessary, treat LAW/UAW infections. In the 1990s, a study conducted with children did not show any correlation between PA in UAW and LAW infections [13]. These results were, however, questioned by a later study that showed identical strains of PA in UAW and LAW in three out of four patients highlighting the need for evaluating treatment at all stages of the disease [14]. Furthermore, there is evidence for a correlation of identical PA and SA strains in the sputum and the bronchoalveolar lavage before and after Ltx suggesting a bidirectional flow between sinuses and LAW in different papers [15–17]. Moreover, there is data on pre-transplant sinus colonization and post-transplant allograft colonization emphasizing that there is an association between the two reservoirs and raising the question how aggressive sinonasal treatment should be, even if patients do not report any complaints [18]. Supportive of the concept of microbial translocation in the CF airway is a study by Pletcher et al. showing a collapse of the niche specificity in CF patients with CRS compared to non-CF CRS patients. Meaning, that the usually distinct microbiome in the lungs and the sinuses merge to form a united airway colonization. Interestingly, the only two lung transplant patients included in that study did not show loss of niche specificity opposing the conclusion of the authors [19]. We previously described that successful control of the sinuses, defined as the presence of three or less sinus aspirates with significant bacterial growth resulted in significant lower incidence of tracheobronchitis and pneumonia [9]. A few years later Shatz et al. reported of aggressive revision sinus surgery in children with CF and a reduction in symptoms and duration of hospitalization as well as intravenous antibiotics and improvement of forced expiratory pressure in 1 s (FEV1). Out of the assessed 15 patients, 3 had a prior Ltx [20]. Similar results of CF patients with revision surgery in patients with not controlled CRS showed a reduced frequency of pulmonary exacerbations requiring hospitalization if treated with modified endoscopic medial maxillectomy [21]. Luparello et al. recently published a study comparing Ltx recipients with and without ESS. The time between Ltx and ESS is not mentioned explicitly. The group with ESS had a significantly higher SNOT-22 score and the indication for ESS was symptom-score-based and not to reclaim UAW before or after transplantation. This contrasts with the concept at our center, where we do not focus on symptom-scores like SNOT-22 but aim to give patients optimal conditions to rinse the nose and prevent chronic colonization of pathognomonic bacteria to protect the allograft. They found a significant difference between pre- and post-ESS forced vital capacity (FVC) at 24 months in the ESS group, whereas the non-ESS group showed no difference pre- and post-transplant [22]. In general these findings align with our results of a significant lower incidence of severe infectious events of the LAW and UAW and a trend to lower incidence in mild infections, which consequently results in fewer antibiotic treatments and hospitalizations and possibly a better function of the allograft [23]. One major difference to our study was the symptom-score-based indication for surgery. Considering that almost all CF patients have pathological or altered sinonasal anatomy and not all of them complain about CRS symptoms, we might rethink the general indication of eESS and reflect a more subjective, patient-centered decision-process as Luparello and colleagues did in their study.

As in previous studies on the same subject there are limitations worth mentioning. First, this is a retrospective, single center study without a randomized control group. We cannot exclude type 2 error in our small population and especially in our non-sinus surgery group. Nevertheless, it is one of the largest series published in current literature. The small cohort prevented us from statistical correction for confounders (rare bacteria such as BCC, co-morbidities, age or sex, cytomegalovirus-status, gastro-intestinal dysfunction). However, there was no difference in age and sex (see Table 1). We included five patients with BCC infection. Three in the sinus surgery group, two in the non-sinus surgery group (see Table 1). Considering the different treatment concepts all over the world in patients with chronic BCC colonization this balanced distribution in both groups seems worth mentioning. In both groups one chronically colonized patient died. To address the above mentioned possible explanation for missing benefit in CLAD-free survival we think there could be a selection bias. There was regular and thorough evaluation of the sinonasal disease by an ear nose throat surgeon which could have biased the selection of patients in both groups. Further analysis of the pre- and postoperative and post-transplant microbiology might contribute additional information for a later study. As mentioned above, the CRP values to screen for infectious events were correlated with clinical documentations and diagnostic procedures. In our institution we heavily rely on the sensitivity of CRP-values for systemic infections after Ltx. However, we acknowledge, that providing more data and a prospective character of a similar study could provide stronger evidence of benefit. This study does not reflect on the evolving field of novel medical agents such as CFTR modulator therapies and its impact on future management of sinus disease in CF patients and improved allograft outcomes. As clinicians experience on a daily basis, the modulator therapies provide a promising conservative treatment option with great impact on symptoms and clinical signs (e.g., polyps) and are already changing current treatment protocols [24].

## Conclusion

The established treatment protocol at our center with early eESS after Ltx is associated with a reduction in severe infectious events of the LAW/UAW. In our analysis, there was no difference in overall survival and CLAD-free survival. We, therefore, encourage clinicians to review a general necessity of eESS in this specific patient cohort. Considering there is no survival nor allograft dysfunction benefit in our data and the sole gain is a reduction in severe infections we advocate to weigh possible risks of eESS against the benefits for patients on an individual basis.

## Data Availability

Data is available upon reasonable request.
